# A case report of a primary pericardial leiomyosarcoma: An extremely rare cardiac neoplasm

**DOI:** 10.1016/j.amsu.2022.103701

**Published:** 2022-04-29

**Authors:** Achraf Machraa, Hanaa El Ghiati, Zineb Fassi Fehri, Cyrille Mbida, Hafsa Chahdi, Fouad Nya, Younes Moutakillah, Zouhair Lakhal, Najat Mouine, Aatif Benyass

**Affiliations:** a Cardiology Center, Mohammed V Military Instruction Hospital of Rabat, Mohammed V University, Morocco; b Department of Pathological Anatomy, Mohammed V Military Instruction Hospital of Rabat, Mohammed V University, Morocco; c Department of Cardiac Surgery, Mohammed V Military Instruction Hospital of Rabat, Mohammed V University, Morocco

**Keywords:** Primary pericardial sarcomas, Leiomyosarcoma, Cardiac tumors, Case report

## Abstract

**Introduction and importance:**

Primary malignant pericardial tumors are an entity that is infrequently encountered and may be a cause of pericardial effusion. Primary pericardial leiomyosarcoma are even rarer, and highly aggressive tumors, with no more than 200 cases reported in the literature. In this case report, we are presenting a rare case of a primary pericardial leiomyosarcoma that was diagnosed at our institution. We discuss the available diagnostic modalities and also shed light on alternative therapies when patients are not ideal surgical candidates.

**Case presentation:**

A 27-year-old male patient was admitted with a gradually worsening dyspnea associated with a deterioration of general condition. Echocardiography examination showed a circumferentiel pericardial effusion with significant fibrin deposits and pericardial thickening. An open surgical biopsy of the pericardium was indicated which was in favor of the diagnosis of pericardial leiomyosarcoma. Unfortunately, the patient died during the procedure.

**Clinical discussion:**

Despite its rarity, primary pericardial leiomyosarcoma should be considered as a differential diagnosis in the assessment of a pericardial effusion of an unknown etiology. Cardiac magnetic resonance imaging is considered to be the reference standard technique for evaluation of a suspected pericardial tumor. Surgical biopsies provide the best odds for detection of the cell of origin, but it's fought with periprocedural risks depending on the site of the tumor.

**Conclusion:**

Primary pericardial leiomyosarcomas appear to have a poor prognosis. Surgical approach is the primary modality of treatment. Chemotherapy and radiotherapy should be offered to patients who are not ideal surgical candidates.

## Highlights

• Pericardial leiomyosarcomas are rare and highly aggressive tumors. The prognosis is poor with 5-year survival rate of 14%.
• Multimodality imaging has enabled identification of the etiology of pericardial tumors in many cases.
• Surgical approach remains the gold standard of the therapy. Chemotherapy and radiotherapy are suitable alternatives.

## 1 Introduction

Cardiac masses are unusual but remain a major component of cardio-oncology clinical practice. These include benign tumors, malignant tumors (primary and secondary) and tumor-like condition. The cardiac involvement can affect the endocardium, myocardium, or pericardium. The prevalence of primary cardiac tumors is reported to be around 0.056–0.02% [1].

Primary pericardial tumors are even rarer, and their prevalence ranges from 0.001 to 0.007% [2], with most pericardial tumors a result of local invasion, hematogenous seeding, or lymphatic spread. Primary pericardial tumors are more frequently malignant and include mesotheliomas, a wide variety of sarcomas, lymphomas amongst which mesotheliomas are the most frequent. Benign pericardial tumors include pericardial cysts, lipomas, paragangliomas, fibromas, hemangiomas.

The advent of multimodality imaging has enabled identification of the etiology of pericardial tumors in many cases, especially in conjunction with information from clinical settings.

In this article, we are presenting a rare case of a primary pericardial leiomyosarcoma that was diagnosed at our institution. We discuss the available diagnostic modalities and also shed light on alternative therapies when patients are not ideal surgical candidates.

This case report has been reported in line with the SCARE Criteria [3].

## 2 Timeline

**Table:** 

Admission	The patient, presenting with gradually worsening dyspnea and a deterioration of general condition, was admitted for suspicion of a pericardial effusion (Chest X-ray showed a symmetrically enlarged cardiac silhouette giving a water bottle configuration)
Day 2	A transthoracic echocardiography (TTE) showed a large circumferential pericardial effusion with significant fibrin deposits and posterior pericardial thickening
One week after admission	Serological tests, immunological tests and tumor markers were negative. The tuberculosis screen was also negative. Pleural fluid analysis revealed a transudative effusion.
Three weeks after admission	An empirical anti-tuberculous therapy was started
Five weeks after admission	The clinical condition of the patient rapidly worsened. He subsequently underwent a surgical biopsy of the pericardium. Unfortunately, he died during the procedure. Histopathological examination of the tissue sample was in favor of the diagnosis of a pericardial leiomyosarcoma.

## 3 Case Presentation

A 27-year-old man, who had no prior medical illness nor drug or family history, is admitted with a gradually worsening shortness of breath on exertion for a few months associated with a nonproductive cough. Also, he was complaining of paroxysmal nocturnal dyspnea, anorexia, and weight loss.

On physical examination: heart rate 120/min, blood pressure 106/70 mmHg, respiratory rate 26/min, and oxygen saturation 98%. Cardiovascular examination showed a raised jugular venous pulse and muffled heart sounds. Respiratory examination showed dullness on percussion with decreased intensity of breath sounds over the right thoracic area.

Laboratory tests found an inflammatory syndrome with a white blood cell count of 19,400/mm3, C-reactive protein was at 146mg/l. Chest X-ray demonstrated an enlarged cardiac silhouette, which was suspicious for a pericardial effusion, with a large right pleural effusion.

**Fig. 1:**
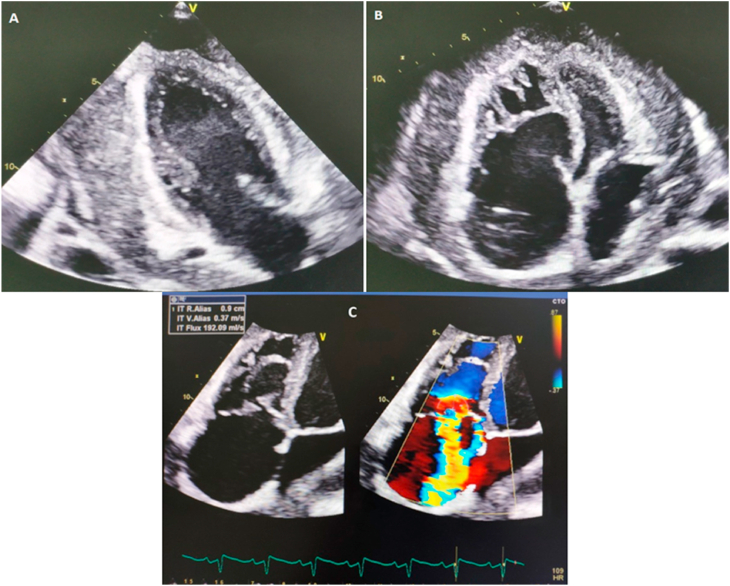
Transthoracic echocardiography showing a large circumferential pericardial effusion with significant fibrin deposits: **(A)** Apical 2-Chamber view, **(B)** Apical 4-Chamber view and **(C)** Severe tricuspid regurgitation.

Transthoracic echocardiography (TTE) confirmed a large circumferential pericardial effusion with significant fibrin deposits filling the pericardial sinuses and recesses and posterior pericardial thickening, without signs of compression. TTE showed also a severe tricuspid regurgitation associated with severe pulmonary hypertension and right ventricular dysfunction (Fig. 1). Pericardiocentesis wasn't feasible due to fibrin deposits.

A possibility of pericardial tuberculosis was considered. However, workup was negative for the presence of acid-fast bacilli in the smear, tuberculosis polymerase chain reaction, and culture. A serology for HIV, HBV and HCV was negative. The immunological tests for common autoimmune cases of serositis and tumor markers were also negative. Pleural fluid analysis revealed a transudative effusion with no bacteria or neoplastic cells.

With the initial assessment being unable to demonstrate the cause of serositis, a computed tomography scan (CT scan) of the chest, abdomen, and pelvis was performed, which showed a circumferential tissue thickening of the pericardium with pleural effusion and ascites.

Based on the clinical features, an empirical anti-tuberculous therapy (Quadtab: 4tablets per day) and treatment with prednisolone (40mg/day) was started. However, the initial outcome was unfavorable since the clinical condition rapidly worsened (weight loss and severe anorexia, polyarthralgia, swelling of the face and right arm, and signs of low cardiac output). A doppler ultrasound of the right upper limb was performed, showing a deep thrombophlebitis of the right subclavian vein extended to the internal jugular vein.

**Fig. 2:**
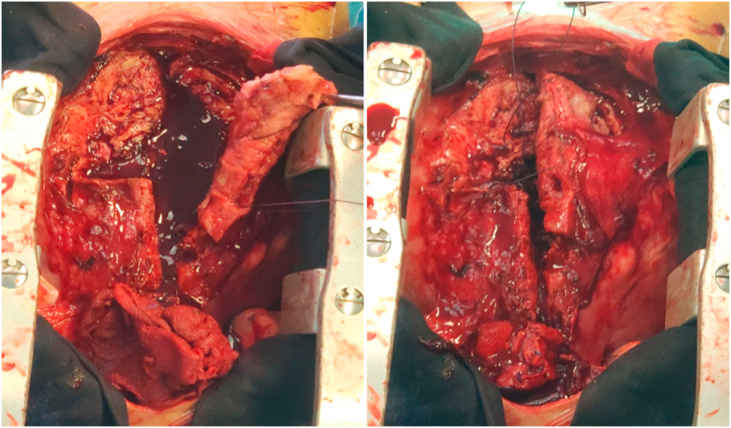
Peroperative image showing an important pericardial thickening, and it was shown to be a pericardial leiomyosarcoma.

A multidisciplinary team meeting, including cardiologists, cardiothoracic surgeons was conducted to decide upon patient management, the team recommended a surgical biopsy of the pericardium (Fig. 2), the procedure was performed, under general anesthesia, by Pr. F. NYA a cardiac surgeon at military hospital of Rabat.

Unfortunately, the patient died during the procedure. Histopathological examination of the tissue sample was in favor of the diagnosis of a pericardial leiomyosarcoma.

## 4 Discussion

Primary pericardial sarcomas are very uncommon and aggressive tumors; they form approximately 13% of all primary cardiac sarcomas [4]. Compared with extracardiac soft tissue sarcomas, cardiac sarcomas are seen in younger patients and have worse prognosis with 5-year survival rate of 14% [5].

Histopathological subtypes of primary pericardial sarcomas include angiosarcomas, leiomyosarcomas, rhabdomyosarcomas, liposarcomas, synovial sarcomas, and undifferentiated pleomorphic sarcomas. Angiosarcomas, followed by leiomyosarcomas, are the most common type of differentiated sarcomas [6].

Leiomyosarcomas are rare but highly aggressive with no more than 200 cases reported in the literature [7]. These tumors are usually seen in the posterior left atrium and present as sessile masses, but it has also been described in other cardiac structures. Like other primary pericardial tumors, pericardial leiomyosarcomas will also have varied clinical presentations often related to pericardial effusion. The presenting symptoms are dyspnea, cough, chest pain, palpitation as well as non-specific symptoms like fatigue, fever, and weight loss. In our case, the main symptom was a gradually progressive dyspnea associated with a deterioration of general condition.

In case of pericardial sarcomas, a TTE is a good initial diagnostic test to detect the associated pericardial effusion or a thickened pericardium. CT scan is useful in the detection of pericardial tumors, the presence of pericardial nodularity is highly suggestive of a pericardial tumor [8].

Modern techniques like multidetector CT scans and cardiac magnetic resonance imaging are gaining favor as the imaging of choice for diagnoses of pericardial sarcomas but their availability is limited, and they are expensive [9].

Surgical biopsies provide the best odds for detection of the cell of origin, but it's fought with periprocedural risks depending on the site of the tumor. Multiple CT-guided biopsies from different accessible sites should be preferred over open surgical biopsies.

In our case, the etiology was not clear at the beginning. The initial assessment provided ambiguous information. Echocardiography examination showed a circumferential pericardial effusion with fibrin deposits and pericardial thickening suggesting the diagnosis of pericardial tuberculosis since tuberculosis is endemic and a major public health problem in our country.

Cardiac magnetic resonance is currently the idead diagnostic imaging technique for assessment and characterization of the pericardium and pericardial masses. It provides multiplanar imaging with a wide field of view, high temporal resolution, and high intrinsic soft tissue contrast without ionizing radiation or iodinated contrast. It also provides information on resectability of masses as well as associated complications such as invasion of mediastinal structures, and encasement of vital structures. Such research has not been done in thi case. The decision to perform an open surgical biopsy of the pericardium was indicated, but the patient died during the procedure.

Surgery is the gold standard of treatment for cardiac sarcomas and effective tumor removal depends on the anatomic location of the tumor. Pericardial leiomyosarcomas may be amenable to removal if detected very early. The response of sarcomas to radiation has been well documented and currently adjuvant radiation is recommended along with surgical resection to improve overall survival [10].

Chemotherapy is reserved to metastatic tumors and the agents of choice are doxorubicin and ifosfamide, with response rates ranging from 55 to 66% [[11], [12]]. In case of pericardial sarcomas, the chemotherapy may be used as the treatment of the primary tumor if surgery is ineffective. The use of doxorubicin may be restricted due to its cardiotoxicity which may confound the mass effects of the tumor and further worsen cardiac reserve. Gemcitabine has shown promise in the treatment of unresectable leiomyosarcoma that have failed anthracycline-based regimen [13]. Further studies are needed to guide chemotherapeutic management.

Primary pericardial leiomyosarcomas appear to have a poor prognosis, and the mean survival without treatment is published to be 6months from the time of diagnosis.

## 5 Conclusion

The relative paucity of such cases makes it difficult to design management protocols specific to pericardial sarcomas. Imaging plays a central role in the evaluation of a suspected tumor, and with multimodality imaging, we are able to accurately distinguish cardiac masses to provide optimal medical management. At this time, surgical approach should be the primary modality of treatment. However, if patients are not found to be ideal surgical candidates, then chemotherapy and radiotherapy should be offered requiring a multidisciplinary approach.

## Sources of funding

The authors declare that this work was not supported by any grants or funding support.

## Ethical approval

The ethical committee approval was not required given the type of the article (Case report). However, the written consent to publish the clinical data of the patient was given and is available to check by the editor of needed.

## Consent for publication

Written informed consent was obtained from the patient for publication of this case report and accompanying images. A copy of the written consent is available for review by the Editor-in-Chief of this journal on request.

## Authors’ contributions

Achraf Machraa was involved in the study concept, the collection of the data, drafting, literature review, and editing of the manuscript.

Hanaa El Ghiati was responsible for literature review and revising the manuscript for important intellectuel content.

Zineb Fassi Fehri was responsible for literature review and revising the manuscript for important intellectuel content.

Cyrille Mbida was responsible for literature review and revising the manuscript for important intellectuel content.

Hafsa Chahdi was responsible for literature review and revising the manuscript for important intellectuel content.

Fouad Nya was responsible of revising the manuscript and data analysis.

Younes Moutakillah was responsible of revising the manuscript and data analysis.

Zouhair Lakhal was responsible of revising the manuscript and data analysis.

Najat Mouine was responsible of data validation and supervision.

Aatif Benyass was responsible of data validation and supervision.

## Registration of research studies

This is not and original research project involving human participants in an interventional or an observational study but a case report, this registration was not required.

## Guarantor

Achraf Machraa.

## Availability of data and materials

The data is available for sharing.

## Provenance and peer review

Not commissioned, externally peer-reviewed.

## Declaration of competing interest

All the authors declare that they have no competing interests.
